# Long-Term Treatment Retention of Levetiracetam Monotherapy in Adults with Epilepsy: A 10-Year Retrospective Cohort Study Using Time-to-Treatment-Failure Analysis

**DOI:** 10.3390/brainsci16101110

**Published:** 2026-10-15

**Authors:** Waad Hasan Alkathiri, Saeed Ali Alqahtani, Abdlatif Ahmed Alghaiheb, Bandar Nasser Aljafen, Bander Balkhi

**Affiliations:** 1 Department of Clinical Pharmacy, College of Pharmacy, King Saud University, P.O. Box 70474, Riyadh 11567, Saudi Arabia; waad-alkathiri@hotmail.com (W.H.A.); saeed@ksu.edu.sa (S.A.A.); alatif@ksu.edu.sa (A.A.A.); 2 College of Medicine, King Saud University, P.O. Box 70474, Riyadh 11567, Saudi Arabia; baljafen@ksu.edu.sa

**Keywords:** levetiracetam, monotherapy, long-term treatment retention, time-to-event analysis, treatment failure, adult epilepsy, RWE

## Abstract

**Highlights:**

**What are the main findings?**

- Levetiracetam monotherapy demonstrated sustained long-term treatment retention as a first-line therapy in adult patients with epilepsy.
- The treatment failure group was younger, had higher renal function, and had predominant generalized-onset seizures.

**What are the implications of the main findings?**

- Our findings support individualized dosing guided by relevant covariates, including age, renal function, and seizure type, to optimize seizure control among patients on levetiracetam monotherapy.
- Future studies are needed to assess the impact of age, seizure types, and renal function on levetiracetam pharmacokinetics and seizure-control outcomes.

**Abstract:**

Background: This study aimed to estimate treatment retention rates for levetiracetam monotherapy in patients with epilepsy and to characterize treatment failure in this population. Methods: A retrospective cohort study included 769 adult patients with epilepsy between 2015 and 2024 at a tertiary hospital in Saudi Arabia who initiated levetiracetam monotherapy. The date of levetiracetam initiation was designated as the index date, and patients were followed from that point until treatment failure or censoring. Treatment failure was defined as either discontinuation of levetiracetam because of adverse events or the addition of another antiseizure medication due to insufficient seizure control. Kaplan–Meier survival analysis was used to estimate the probability of remaining on levetiracetam monotherapy over the follow-up period. Differences in patient characteristics were assessed between the treatment-failure and success groups using Student’s *t*-test for continuous variables and either the chi-square test or Fisher’s exact test for categorical variables. Results: Of the 769 patients who initiated levetiracetam monotherapy, 143 (18.55%) experienced treatment failure during the 10-year follow-up period. The overall mean time to levetiracetam treatment failure was 93.58 months. The 25th percentile time to treatment failure was 95 months (95% CI: 60.167–103.133). The median time to treatment failure was not reached. Females comprised 50.3% of the cohort, with no significant difference in sex distribution between groups (*p* = 0.136). Patients in the treatment-failure group were younger (39.85 ± 16.88 vs. 45.76 ± 19.48, *p* = 0.0003), had higher estimated Creatinine clearance (CrCl) (132.19 ± 57.83 vs. 118.50 ± 61.15, *p* = 0.0150), and had predominantly generalized seizure onset (40.56% vs. 33.71%, *p* = 0.0182) compared with the treatment-success group. Conclusions: Levetiracetam monotherapy demonstrated sustained long-term treatment retention when used as first-line therapy in adults with epilepsy.

## 1. Introduction

Epilepsy is one of the most common neurological disorders, affecting approximately 50 million people worldwide [1]. Its prevalence rate in Saudi Arabia has been estimated at 6.54 per 1000 individuals [2]. Epilepsy is characterized by recurrent seizure episodes; a patient will be diagnosed with epilepsy if an individual has two or more unprovoked seizure episodes that occur more than 24 h apart. A seizure is abnormal electrical activity in the brain that causes behavioral alteration. An uncontrolled seizure is a life-threatening condition that may lead to stroke and permanent disability [3,4].

The burden of epilepsy has decreased in part due to the availability of effective antiseizure medications [5,6]. The efficacy and safety of antiseizure medications vary with their mechanisms of action [7,8]. Long-term use of antiseizure medications has various adverse effects [9,10,11].

Levetiracetam is well tolerated and has broad-spectrum efficacy in treating various types of epilepsy and a favorable safety profile with fewer adverse events. Initially, levetiracetam was used as an adjunct to antiseizure medications to control seizure peaks in the resistant types of epilepsy. In both adults and children, adjunctive levetiracetam reduced seizure frequency more effectively than placebo in patients with focal epilepsy. Pooled results showed that levetiracetam more than doubled the likelihood of achieving at least a 50% reduction in seizure frequency [12,13]. Adjunctive Levetiracetam provides effective and sustained seizure control in generalized epilepsy, with over half of patients achieving prolonged seizure freedom and an overall favorable tolerability profile [14]. Levetiracetam is not significantly metabolized by the cytochrome P450 enzyme system, which reduces the potential for pharmacokinetic drug–drug interactions with other antiseizure medications [15]. Based on these findings, adjunctive levetiracetam is a preferred add-on therapy for inadequately controlled seizures and is among the most favorable agents for combination therapy. More recently, levetiracetam has shown efficacy as first-line monotherapy in various types of epilepsy, including focal and idiopathic generalized epilepsies, particularly those characterized by myoclonic seizures, women of childbearing potential, and older adults [16,17,18]. Due to its unique mechanism of action, it has a favorable safety profile with fewer adverse events. Levetiracetam is a preferred antiseizure medication for women of childbearing age with idiopathic generalized epilepsy, particularly in juvenile myoclonic epilepsy [19,20].

Although levetiracetam monotherapy is widely used for epilepsy, evidence on its long-term treatment retention beyond 5 years in routine real-world practice remains limited. Data describing treatment retention and failure over extended periods, particularly in Saudi populations, are limited. We aimed to estimate the treatment retention of Levetiracetam monotherapy in patients with epilepsy and to define the characteristics of treatment failure of levetiracetam monotherapy in patients with epilepsy.

## 2. Materials and Methods

### 2.1. Study Design, Setting and Population

This retrospective observational cohort study evaluated time to treatment failure with levetiracetam monotherapy over a 10-year study period. Individual follow-up began on the index date and continued until treatment failure, loss to follow-up, or administrative censoring at the end of the study, whichever occurred first. It was conducted at King Saud University Medical City (KSUMC), a major tertiary teaching hospital in Riyadh, Saudi Arabia. The study included all patients who met the inclusion and exclusion criteria during the study period. Inclusion criteria: Adult patients aged 18 or older who have been diagnosed with epilepsy and initiated levetiracetam as monotherapy. Exclusion criterion: Patients who have fewer than two levetiracetam prescriptions within 12 months.

### 2.2. Data Source

A retrospective chart review was conducted via the Electronic Medical Record (EMR) at KSUMC. The study period began on 1 January 2015, and ended on 31 December 2024. First, to identify patient diagnoses with epilepsy during the study period, EMR extraction was based on the International Classification of Diseases, Tenth Revision (ICD-10) codes G40 and G41, and any occurrence of the terms “epilepsy” or “seizure” within the diagnosis free-text field was included. Then a chart review was conducted to extract variables (levetiracetam prescription date, dispensed amount, brand name, dose, patient age, gender, serum creatinine, weight, height, outcome status, seizure type, etiology of epilepsy, and duration after epilepsy diagnosis). Seizure onset was classified according to the 2017 International League Against Epilepsy (ILAE) operational classification as focal, generalized, or unknown onset [21]. Unknown-onset seizures were classified according to the treating neurologist’s documented diagnosis. The Levetiracetam dose was defined as the total daily dose (mg/day) documented immediately before treatment failure among patients who experienced the outcome, or at the last available follow-up among patients who were censored. Data cleaning included exclusion of individuals aged <18 years and those initiating levetiracetam in combination with other antiseizure medications or patients who had received any antiseizure medication before initiating levetiracetam. Key variables (index date, exposure, and outcome) were validated, and implausible records were excluded. Renal function was assessed using creatinine clearance (CrCl), estimated using the Cockcroft–Gault equation and expressed in mL/min. Missing data and outliers were assessed and handled appropriately. Potential outliers were reviewed against the original medical records. Values confirmed to result from data-entry or data-extraction errors were corrected based on the source data, whereas clinically plausible extreme values were retained in the analysis. For continuous variables (weight, height and serum creatinine) with missing observations, single imputation was performed using the mean for approximately normally distributed variables.

### 2.3. Outcome Definition

The primary outcome was time to treatment failure of levetiracetam, which was measured as the number of days without treatment failure, defined as the period from initiation to the first treatment failure. Because individual seizure frequency and seizure-freedom data were not consistently available, the study evaluated treatment retention rather than direct seizure control. Remaining free of treatment failure should not be interpreted as seizure freedom. The index date was defined as the date of the first levetiracetam prescription during the study window. Levetiracetam treatment failure is defined as discontinuation due to an adverse event or the addition of other antiseizure medications to the management plan due to poor seizure control. Poor seizure control was defined as uncontrolled seizures despite documented adherence to levetiracetam monotherapy, as recorded in the treating epileptology clinical notes. Treatment failures were verified by chart review. Censored is defined as loss to follow-up or reaching the study end date of 31 December 2024.

### 2.4. Statistical Analysis

Descriptive statistics were used to summarize the cohort, with frequencies and percentages for categorical variables and means ± standard deviations (SD) for continuous variables. Given the large sample size, continuous variables were assessed using visual inspection of histograms and Shapiro–Wilk testing. The distributions were considered sufficiently symmetrical to justify parametric comparisons.

Patient characteristics were compared between patients with and without treatment failure using Student’s *t*-test for continuous variables and chi-square test or Fisher’s exact test for categorical variables.

Time-to-event analyses were performed using the Kaplan–Meier method to estimate the cumulative probability of Levetiracetam treatment retention over follow-up, defined as remaining free of treatment failure, with 95% confidence intervals. Mean time to treatment failure was estimated from the area under the Kaplan–Meier treatment-failure-free curve over the observed follow-up period. Differences between strata were evaluated using the log-rank test. Associations between covariates and survival were assessed using univariable Cox proportional hazards regression. Multivariable Cox proportional hazards models were used to estimate the adjusted associations of covariates with survival. Results are reported as adjusted hazard ratios (aHRs) with 95% confidence intervals (CIs). The proportional hazards assumption was evaluated using the supremum test. Among models satisfying this assumption, model fit was compared using the Akaike information criterion (AIC), with lower AIC values indicating better fit. Final model selection was based on AIC and the clinical relevance of the covariates. The *p*-value of less than 0.05 was considered to indicate statistical significance. Data cleaning and statistical analyses were conducted using SAS software, version 9.4 (SAS Institute, Cary, NC, USA).

### 2.5. Bias Reduction Measures

A sensitivity analysis was conducted to assess the potential influence of the prescription-based exclusion criterion. Patients excluded from the primary cohort because they had fewer than two levetiracetam prescriptions within 12 months were eligible for inclusion in the sensitivity analysis if evaluable post-index dispensing or clinical follow-up was available. Kaplan–Meier estimation and Cox proportional hazards regression were repeated in the expanded cohort, and the results were compared with those of the primary analysis.

## 3. Results

**Figure 1:**
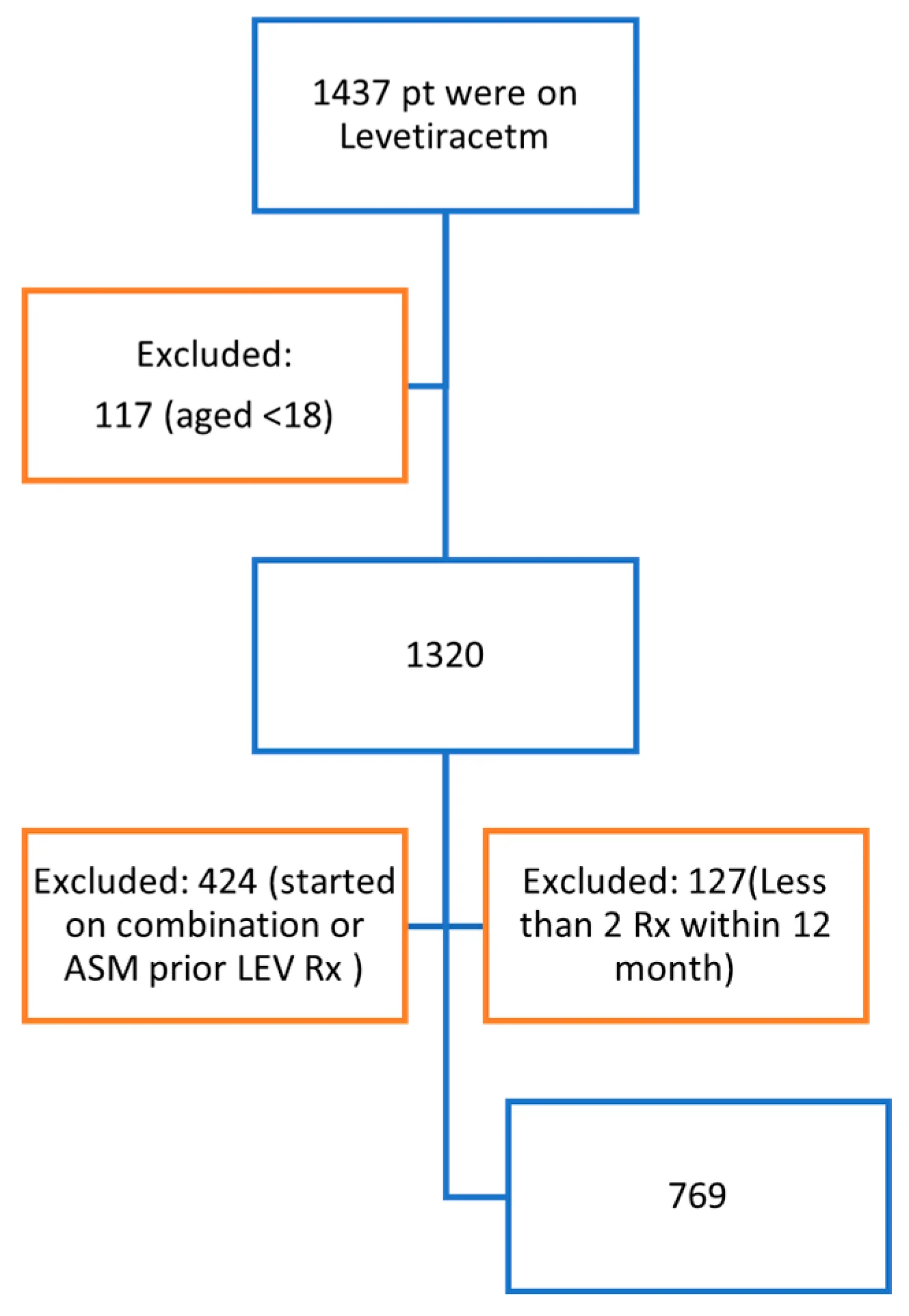
Cohort flow diagram.

A total of 1437 patients were identified. Of these, 117 were excluded because they were aged <18 years, 424 because of prior antiseizure medication use or initiation of levetiracetam as combination therapy, and 127 because they had fewer than two levetiracetam prescriptions within 12 months. The final cohort included 769 patients (Figure 1).

The mean age was 44.66 (±19.48) years. 50.33% were female; however, sex distribution did not differ significantly between groups (*p* = 0.136). Seizure types were classified as Focal (20.94%), Generalized (34.98%), and Unknown onset (44.08%). The mean duration of epilepsy since diagnosis was 6.83 (±7.49) years (Table 1).

### 3.1. Primary Outcome—Levetiracetam Time to Treatment Failure

Over the 10-year follow-up period, 143 (18.55%) patients experienced levetiracetam treatment failure, while 626 (81.40%) remained free from documented treatment failure during the observed follow-up. Of these, 600 completed follow-up without a treatment-failure event, whereas 26 were lost to follow-up and were censored at their last documented visit. Among those who experienced treatment failure, 137 (95.80%) were classified as poor responders to levetiracetam monotherapy, whereas only 6 (4.20%) failed treatment due to intolerance. Adverse events leading to levetiracetam discontinuation included low mood and depressive symptoms in two patients, sedation in one, aggression with agitation in one, neutropenia in one, and gastrointestinal upset in one.

Time to Levetiracetam treatment failure was analyzed. The mean follow-up was 48.75 months (SD 34.78). The median Levetiracetam dose was 1250 mg/day (IQR 250–4000 mg). The overall mean time to Levetiracetam treatment failure was 93.58 months. The 25th percentile time to Levetiracetam treatment failure was 95 months (95% CI: 60.167–103.133). However, the late estimate was based on a smaller risk set. The median time to treatment failure was not reached. The mean time to levetiracetam discontinuation due to adverse events was 26.90 (SD 38.64) months.

**Figure 2:**
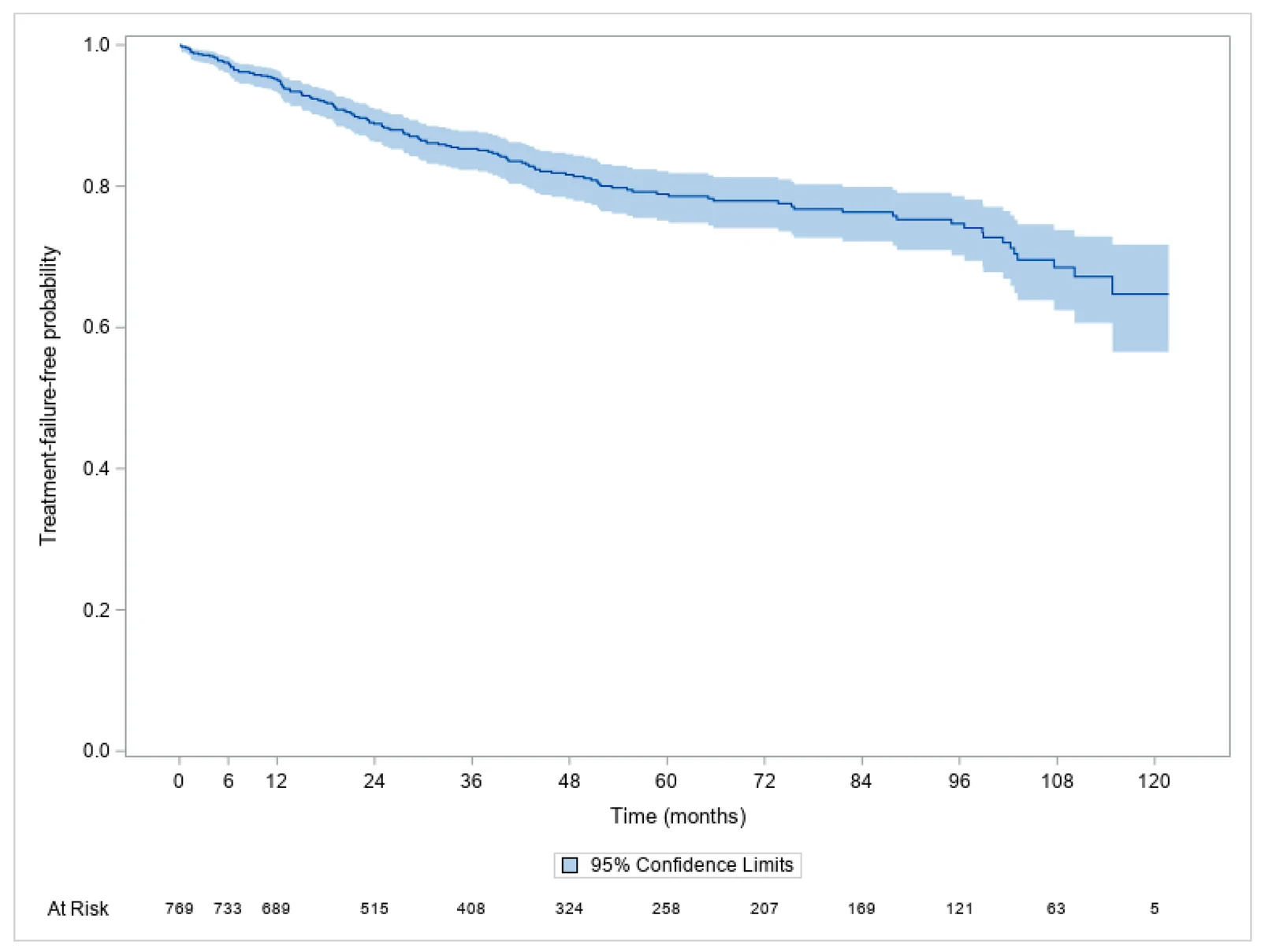
Kaplan–Meier curve showing time to treatment failure after initiation of Levetiracetam monotherapy. Shaded areas represent 95% confidence intervals. Numbers at risk are shown below the curve.

The Kaplan–Meier-estimated treatment-failure-free probability was 97.37% (95% CI, 95.9–98.2%) at 6 months, 94.93% (95% CI, 93.1–96.2%) at 12 months, 88.48% (95% CI, 85.8–90.6%) at 24 months, 78.89% (95% CI, 75.1–82.1%) at 5 years, and 64.69% (95% CI, 56.5–71.7%) at 10 years (Figure 2).

### 3.2. Secondary Outcome: Characteristics of the Treatment-Failure Group

Compared with the treatment-success group, patients in the treatment-failure group were younger (39.85 ± 16.88 vs. 45.76 ± 19.48 years; *p* < 0.001), more frequently received doses ≥1500 mg/day (70.63% vs. 45.21%; *p* < 0.001), had higher estimated creatinine clearance (132.19 ± 57.83 vs. 118.50 ± 61.15 mL/min; *p* = 0.015), and more frequently had generalized-onset seizures (40.56% vs. 33.71%; overall *p* = 0.018 for seizure-onset distri-bution).

In the subgroup analysis, Kaplan–Meier analysis stratified by age group showed higher treatment-failure-free probabilities among older patients (aged ≥65 years) during follow-up (log-rank *p* = 0.0359). Kaplan–Meier analysis of the 12 strata defined by levetiracetam dose, age, and renal function showed significant differences in treatment-failure-free probability (log-rank *p* = 0.0003), with the highest probability observed among patients aged ≥65 years receiving lower doses with impaired renal function (Crcl < 90) and the lowest among patients aged <65 years receiving high doses with augmented renal clearance (Crcl ≥ 130) (Supplementary Figure S1).

**Figure 3:**
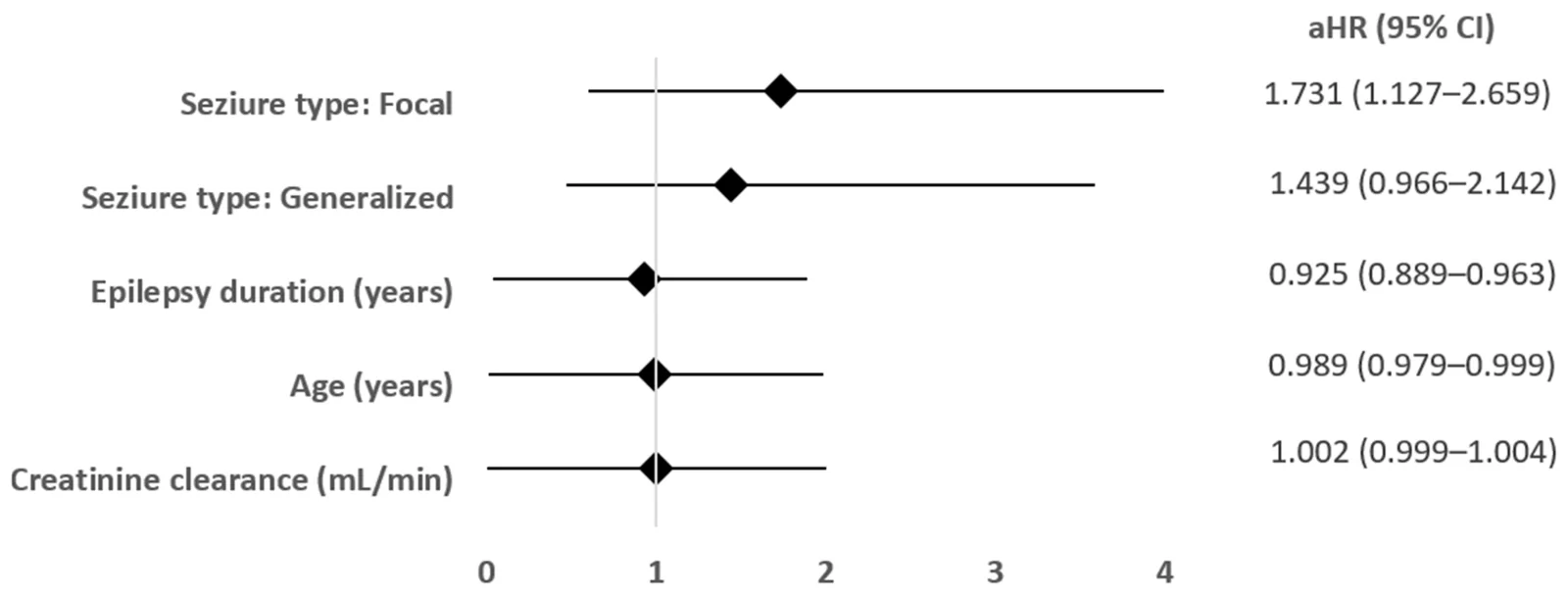
Factors associated with Levetiracetam treatment failure. Forest plot showing adjusted hazard ratios (aHRs) and 95% confidence interval (95% CI) from the multivariable Cox proportional hazards model. An aHR >1 indicates a higher hazard of levetiracetam treatment failure, whereas an aHR <1 indicates a lower hazard. The vertical line at aHR = 1 represents no association.

In the multivariable Cox proportional hazards model, age, seizure type, and epilepsy duration were independently associated with levetiracetam treatment failure. Each additional year of age was associated with approximately a 1.1% lower hazard of levetiracetam treatment failure (aHR = 0.989; 95% CI = 0.979–0.999; *p* = 0.033). Compared with patients with unknown seizure onset, patients with focal seizure onset had a 73% higher hazard of levetiracetam treatment failure (aHR = 1.731; 95% CI = 1.127–2.659; *p* = 0.012). Each additional year of epilepsy duration is associated with approximately a 7.5% lower hazard of levetiracetam treatment failure (aHR = 0.925; 95%CI = 0.889–0.963; *p* = 0.0001). While each ml/min increase in Creatinine clearance was associated with a 0.2% higher hazard of levetiracetam treatment failure, this was not statistically significant (aHR = 1.002; 95% CI = 0.999–1.004; *p* = 0.156). Factors associated with Levetiracetam treatment failure are described in Figure 3.

### 3.3. Sensitivity Analysis

Of the 127 patients excluded from the primary cohort because they had fewer than two levetiracetam prescriptions within 12 months, 106 had no documented subsequent levetiracetam dispensing or clinical follow-up and therefore had no evaluable post-index observation time. These patients could not contribute valid time-to-event information. The remaining 21 patients had sufficient post-index follow-up and were added to the primary cohort, resulting in an expanded cohort of 790 patients. Kaplan–Meier treatment-retention estimates and adjusted hazard-ratio estimates in the expanded cohort were similar in direction and magnitude to those obtained in the primary analysis. Nevertheless, because 106 excluded patients lacked evaluable post-index follow-up, residual selection bias associated with the prescription-based eligibility criterion could not be excluded.

## 4. Discussion

In clinical practice, antiseizure medication utilization is a pragmatic, patient-centered outcome that integrates sustained effectiveness, tolerability, and treatment acceptability over time. Long-term levetiracetam retention over a maximum follow-up of 10 years using time-to-treatment-failure analysis in an adult cohort with a mean follow-up of 48.75 months. Our study addresses a key evidence gap by providing extended real-world evidence that complements the shorter follow-up periods typically available in randomized clinical trials and meta-analyses.

Levetiracetam demonstrated durable long-term retention, with the estimated 25th percentile of time to treatment failure occurring at 95 months (approximately 8 years). Treatment retention is a composite measure reflecting effectiveness, tolerability, prescribing decisions, patient adherence, and healthcare access; therefore, it should not be interpreted as a direct measure of antiseizure efficacy. Nevertheless, retention remains a clinically meaningful real-world outcome because it captures the overall success of long-term therapy in routine practice.

The estimated five-year retention probability in our cohort was higher than that reported by Tabrizi et al. Direct comparison should be interpreted cautiously because the studies differed in patient selection, epilepsy classification, treatment setting, and outcome definition. The Tabrizi cohort focused on idiopathic generalized epilepsy, whereas our cohort included focal, generalized, and unknown-onset seizures. In addition, differences in the handling of prescription gaps, treatment modification, loss to follow-up, and discontinuation may have contributed to the higher retention estimate observed in our study [22].

Our findings align with previous literature on the long-term effectiveness of levetiracetam monotherapy. Randomized controlled trials have shown that levetiracetam is non-inferior to carbamazepine and valproate for initial monotherapy in focal and generalized epilepsy, with 6- to 12-month seizure freedom rates ranging from 60% to 75% in newly diagnosed populations [16]. Notably, the treatment failure rate observed in our study (18.55%) is broadly consistent with prior observational studies reporting discontinuation or failure rates of 15–30% over 1–2 years, although few studies have extended outcome assessment beyond 5 years [23]. More recently, a Swedish nationwide registry study showed higher 5-year levetiracetam retention among adults aged ≥50 years than among those aged 18–49 years (63.5% vs. 45.3%), similar to our cohort and supporting the potential influence of age on long-term treatment retention [24].

In this cohort, patients who experienced treatment failure were younger, were more likely to have normal or augmented renal clearance, and received higher levetiracetam doses. Patients aged <65 years showed lower treatment-failure-free probabilities; this could be explained by the younger population having better renal function, leading to faster elimination of levetiracetam and lower concentrations, which may require higher doses to compensate for a shorter half-life and achieve optimal seizure control [25]. Renal function may influence levetiracetam clearance and, consequently, serum concentrations. Previous pharmacokinetic studies have demonstrated lower trough concentrations and reduced overall exposure with increasing CrCl, particularly in patients with augmented renal clearance. Conversely, impaired renal function is associated with reduced levetiracetam clearance and greater drug exposure [26]. Levetiracetam dose escalation was guided by clinical response and seizure frequency rather than therapeutic drug concentration targets [25]. However, only the total daily levetiracetam dose documented immediately before treatment failure or censoring was captured. Information on initial and maintenance doses, longitudinal dose escalation, and renal function-guided dose modifications was not available. Thus, higher doses may reflect dose escalation in patients with inadequate seizure control and may therefore represent confounding by indication, whereby patients with more severe cases are more likely to receive higher doses. Consequently, dose was considered a post-baseline variable and was not included in the multivariable Cox proportional hazards model. Accordingly, the association between higher levetiracetam doses and treatment failure should therefore be interpreted cautiously.

Subgroup analyses showed that 40.89% of patients receiving higher doses had augmented renal clearance (CrCl ≥ 130 mL/min) (Supplementary Table S2), whereas 45.45% of those receiving lower doses had impaired renal function (CrCl < 90 mL/min). Kaplan–Meier analyses further showed the highest treatment-failure-free probability among patients aged ≥65 years receiving lower doses with impaired renal function and the lowest among patients aged <65 years receiving higher doses with augmented renal clearance. This pattern may reflect age- and renal function-related differences in levetiracetam pharmacokinetics, particularly drug clearance, and warrants further pharmacokinetic and pharmacodynamic studies incorporating longitudinal dosing, serum concentrations, renal function, and clinical response.

Seizures of generalized onset predominated among patients who experienced levetiracetam treatment failure; Kaplan–Meier curves suggested higher treatment-failure-free probabilities among patients with unknown-onset seizures, whereas the curves for focal- and generalized-onset seizures largely overlapped. However, the overall Kaplan–Meier comparison did not demonstrate a statistically significant difference among seizure-onset groups. In the adjusted Cox model, focal-onset seizures were associated with a 73% higher hazard of treatment failure relative to the unknown-onset reference group, but not relative to the generalized-onset group. In this cohort, seizure onset was classified according to the diagnosis documented by the treating neurologist. The unknown-onset category included patients for whom a definitive focal or generalized onset classification was not documented in the available medical record. Given the large and potentially heterogeneous unknown-onset category, which comprised approximately 44.08% of the cohort, these subgroup findings should be regarded as exploratory and should not be interpreted as demonstrating differential levetiracetam effectiveness by seizure onset.

The apparent protective association between longer epilepsy duration and levetiracetam treatment failure may reflect a survivor effect, whereby patients with longer disease duration may represent those with a less severe disease course. In contrast, patients with more severe epilepsy may experience treatment failure earlier.

Although the relationship between levetiracetam concentration and seizure control remains uncertain, our findings support individualized dosing informed by relevant clinical covariates, including age, renal function, and seizure type, while recognizing that therapeutic drug monitoring and prospective pharmacokinetic data were unavailable in this cohort. To our knowledge, this is among the largest Middle Eastern cohorts evaluating levetiracetam monotherapy retention and among the few studies reporting follow-up extending to 10 years in routine clinical practice. The large sample size and extended observation period provide valuable real-world evidence regarding long-term treatment retention.

Several limitations should be acknowledged. First, the single-center design may restrict the external validity of the findings, particularly across healthcare systems with different patient populations and prescribing practices. Second, the absence of a comparator group precludes concluding the relative effectiveness of levetiracetam compared with other antiseizure medications. Third, time to treatment failure was used as a pragmatic measure of real-world levetiracetam treatment effectiveness. However, detailed longitudinal data on seizure freedom, time to seizure control, time to seizure recurrence, and seizure status at predefined follow-up intervals were not consistently available in this cohort. Treatment retention therefore does not directly quantify seizure control and may also be influenced by adherence, patient preferences, access to care, and clinician prescribing decisions. Accordingly, treatment-failure-free probabilities should not be interpreted as direct estimates of seizure freedom. Moreover, Kaplan–Meier analysis assumes non-informative censoring, and loss to follow-up related to treatment response may have biased the long-term estimates. Although follow-up extended to 10 years, the progressively smaller risk set beyond approximately 8 years reduced the precision of the late Kaplan–Meier estimates; therefore, the 10-year treatment-failure-free probability should be interpreted cautiously. Finally, consider the retrospective design, residual confounding, incomplete documentation, especially of adverse events, and changes in clinical practice over the 10-year observation period when interpreting the findings. Future studies should incorporate standardized ILAE seizure classification to reduce the proportion of unknown-onset seizures and improve precision of seizure subtype analyses.

## 5. Conclusions

Levetiracetam monotherapy demonstrated sustained long-term treatment retention when used as first-line therapy in adults with epilepsy. Further studies are warranted to evaluate the impact of age, seizure type, and renal function on levetiracetam pharmacokinetics and seizure-control outcomes.

## Figures and Tables

**Table 1:** Patients’ characteristics stratified by outcome status. Baseline characteristics of patients according to treatment outcome.

Characteristic	Overall (N = 769)	Treatment Success (n = 626)	Treatment Failure (n = 143)	*p* Value
**Age, years, mean ± SD**	44.66 ± 19.48	45.76 ± 19.88	39.85 ± 16.87	**<0.001**
**Sex, n (%)**				0.136
Female	387 (50.33)	307 (49.04)	80 (55.94)	
Male	382 (49.67)	319 (50.96)	63 (44.06)	
**Seizure onset, n (%)**				**0.018**
Focal onset	161 (20.94)	124 (19.81)	37 (25.87)	
Generalized onset	269 (34.98)	211 (33.71)	58 (40.56)	
Unknown onset	339 (44.08)	291 (46.49)	48 (33.57)	
**Epilepsy etiology, n (%)**				0.364
Structural	392 (50.98)	324 (51.76)	68 (47.55)	
Non-structural	377 (49.02)	302 (48.24)	75 (52.45)	
**Epilepsy duration(years) mean ± SD**	6.83 ± 7.49	6.95 ± 7.05	6.31 ± 9.16	0.439
**BMI category, n (%)**				0.162
Underweight	52 (6.76)	41 (6.55)	11 (7.69)	
Normal weight	214 (27.83)	174 (27.80)	40 (27.97)	
Overweight	244 (31.73)	209 (33.39)	35 (24.48)	
Obesity	259 (33.68)	202 (32.27)	57 (39.86)	
**Serum creatinine, µmol/L, mean ± SD**	83.39 ± 78.12	85.39 ± 81.46	74.64 ± 60.87	0.076
**Creatinine clearance, mL/min, mean ± SD**	121.05 ± 60.74	118.50 ± 61.15	132.19 ± 57.83	**0.015**
**Creatinine clearance category, n (%)**				**0.0068**
≥130 mL/min	281 (36.54)	221 (35.30)	60 (41.96)	
90–<130 mL/min	259 (33.68)	205 (32.75)	54 (37.76)	
<**90** mL/min	229 (29.78)	200 (31.95)	29 (20.28)	
**Levetiracetam dose, n (%)**				**<0.001**
<1500 mg/day	385 (50.07)	343 (54.79)	42 (29.37)	
≥1500 mg/day	384 (49.93)	283 (45.21)	101 (70.63)	
**Neurological comorbidities, n (%)**				
Stroke	113 (14.69)	103 (16.45)	10 (6.99)	0.004
Multiple sclerosis	19 (2.47)	14 (2.24)	5 (3.50)	0.381
Cerebral palsy	3 (0.39)	2 (0.32)	1 (0.70)	0.511
Migraine	8 (1.04)	4 (0.64)	4 (2.80)	0.022
ADHD	4 (0.52)	3 (0.48)	1 (0.70)	0.741
Mental illness	23 (2.99)	17 (2.72)	6 (4.20)	0.349
Alzheimer disease	7 (0.91)	5 (0.80)	2 (1.40)	0.496
Other neurological comorbidities	12 (1.56)	8 (1.28)	4 (2.80)	0.186
**Psychiatric comorbidity, n (%)**				0.399
One condition	33 (4.29)	24 (3.83)	9 (6.29)	
Two conditions	4 (0.52)	3 (0.48)	1 (0.70)	

Abbreviations: ADHD, attention-deficit/hyperactivity disorder; BMI, body mass index; CrCl, creatinine clearance; SD, standard deviation. Notes: Data are presented as n (%) unless otherwise indicated. Percentages are calculated within columns. Continuous variables are presented as mean ± SD. Creatinine clearance was calculated using the Cockcroft–Gault equation. *p* values were derived using appropriate statistical tests for continuous and categorical variables. Bold *p* values indicate statistical significance (*p* < 0.05).

## Data Availability

The data presented in this study are available on request from the corresponding author. The data are not publicly available due to patient privacy, confidentiality, and ethical considerations.

## Supplementary Materials

The following supporting information can be downloaded at: https://www.mdpi.com/article/10.3390/brainsci16101110/s1, Figure S1: Kaplan–Meier curves of treatment-failure-free probability stratified by prespecified clinical variables and combined clinical strata; Table S1: STROBE checklist for reporting the retrospective cohort study; Table S2: Distribution of Levetiracetam Dose Categories Across Renal Function Groups.
