# Supplementary material for: Long-Term Treatment Retention of Levetiracetam Monotherapy in Adults with Epilepsy: A 10-Year Retrospective Cohort Study Using Time-to-Treatment-Failure Analysis
Source: Brain Sci. 2026 Oct 15;16(10):1110. doi: 10.3390/brainsci16101110 (PMC13702674; doi:10.3390/brainsci16101110)
Supplement: Supplementary file 1 [file brainsci-16-01110-s001.zip › brainsci-4568865-supplementary.pdf]

Supplementary Table S1: STROBE Statement—Checklist of items that should be included in reports of cohort studies

	Item No	Recommendation	Page No.
**Title and abstract**	1	(*a*) Indicate the study's design with a commonly used term in the title or the abstract	1
		(*b*) Provide in the abstract an informative and balanced summary of what was done and what was found	1-2
**Introduction**			
Background/rationale	2	Explain the scientific background and rationale for the investigation being reported	2-3
Objectives	3	State specific objectives, including any prespecified hypotheses	3
**Methods**			
Study design	4	Present key elements of study design early in the paper	3
Setting	5	Describe the setting, locations, and relevant dates, including periods of recruitment, exposure, follow-up, and data collection	3
Participants	6	(*a*) Give the eligibility criteria, and the sources and methods of selection of participants. Describe methods of follow-up	3
		(*b*) For matched studies, give matching criteria and number of exposed and unexposed	
Variables	7	Clearly define all outcomes, exposures, predictors, potential confounders, and effect modifiers. Give diagnostic criteria, if applicable	3-4
Data sources/ measurement	8*	For each variable of interest, give sources of data and details of methods of assessment (measurement). Describe comparability of assessment methods if there is more than one group	*3*
Bias	9	Describe any efforts to address potential sources of bias	4
Study size	10	Explain how the study size was arrived at	3
Quantitative variables	11	Explain how quantitative variables were handled in the analyses. If applicable, describe which groupings were chosen and why	3
Statistical methods	12	(*a*) Describe all statistical methods, including those used to control for confounding	4
		(*b*) Describe any methods used to examine subgroups and interactions	4
		(*c*) Explain how missing data were addressed	3
		(*d*) If applicable, explain how loss to follow-up was addressed	3
		(*e*) Describe any sensitivity analyses	4
**Results**			
Participants	13*	(a) Report numbers of individuals at each stage of study—eg numbers potentially eligible, examined for eligibility, confirmed eligible, included in the study, completing follow-up, and analysed	4-5
		(b) Give reasons for non-participation at each stage	5
		(c) Consider use of a flow diagram	5
Descriptive data	14*	(a) Give characteristics of study participants (eg demographic, clinical, social) and information on exposures and potential confounders	5
		(b) Indicate number of participants with missing data for each variable of interest	5
		(c) Summarise follow-up time (eg, average and total amount)	6
Outcome data	15*	Report numbers of outcome events or summary measures over time	6
Main results	16	(*a*) Give unadjusted estimates and, if applicable, confounder-adjusted	6-7

		estimates and their precision (eg, 95% confidence interval). Make clear which confounders were adjusted for and why they were included	
		(*b*) Report category boundaries when continuous variables were categorized	5-6
		(*c*) If relevant, consider translating estimates of relative risk into absolute risk for a meaningful time period	
Other analyses	17	Report other analyses done—eg analyses of subgroups and interactions, and sensitivity analyses	7
**Discussion**			
Key results	18	Summarise key results with reference to study objectives	8
Limitations	19	Discuss limitations of the study, taking into account sources of potential bias or imprecision. Discuss both direction and magnitude of any potential bias	10
Interpretation	20	Give a cautious overall interpretation of results considering objectives, limitations, multiplicity of analyses, results from similar studies, and other relevant evidence	9-10
Generalisability	21	Discuss the generalisability (external validity) of the study results	9
**Other information**			
Funding	22	Give the source of funding and the role of the funders for the present study and, if applicable, for the original study on which the present article is based	11

*Give information separately for exposed and unexposed groups.

Note: An Explanation and Elaboration article discusses each checklist item and gives methodological background and published examples of transparent reporting. The STROBE checklist is best used in conjunction with this article (freely available on the Web sites of PLoS Medicine at http://www.plosmedicine.org/, Annals of Internal Medicine at http://www.annals.org/, and Epidemiology at http://www.epidem.com/). Information on the STROBE Initiative is available at http://www.strobe-statement.org.

Supplementary Table S2. Distribution of Levetiracetam Dose Categories Across Renal Function Groups

Levetiracetam dose category	CrCl <90 mL/min, n (%)	CrCl 90–129 mL/min, n (%)	CrCl ≥130 mL/min, n (%)	Total, n (%)
**Low dose** (<1500 mg/day)	175 (45.45)	110 (28.57)	100 (25.97)	385
**High dose** (≥1500 mg/day)	101 (26.30)	126 (32.81)	157 (40.89)	384
**Total**	276	236	257	769
**CrCl,** Creatinine Clearance, **%, Raw percentage**				

Supplementary Figure S1. Kaplan–Meier Subgroup Analyses of Treatment-Failure-Free Probability Across Individual and Combined Clinical Strata

A1

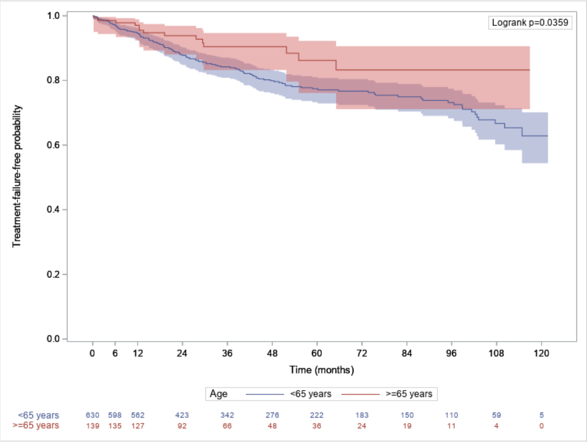

A2

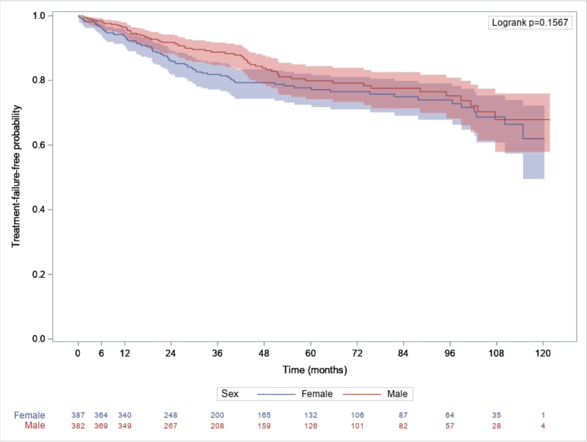

A3

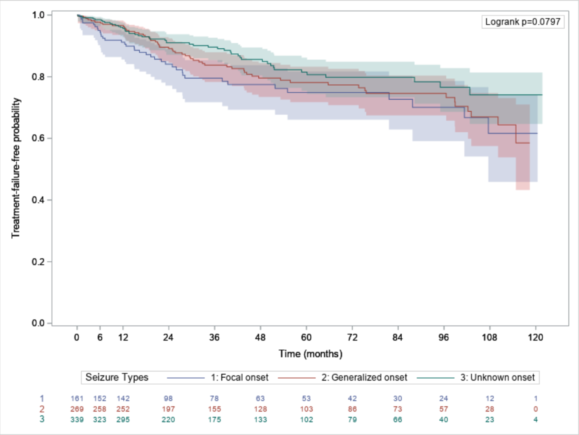

A4

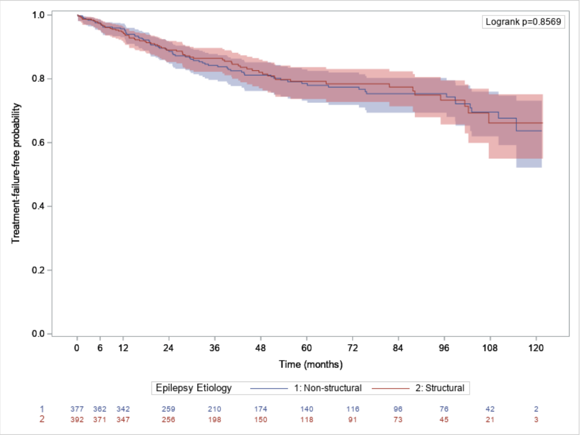

A5

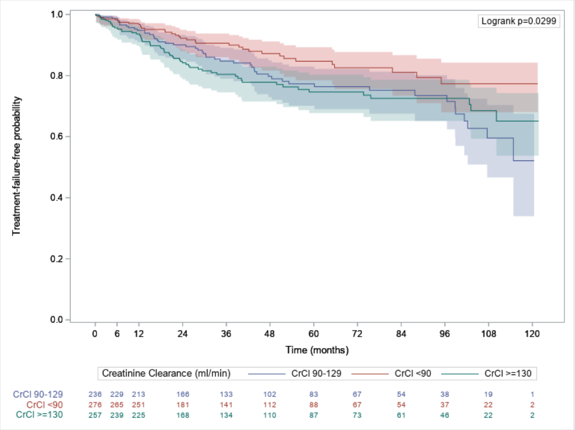

A6

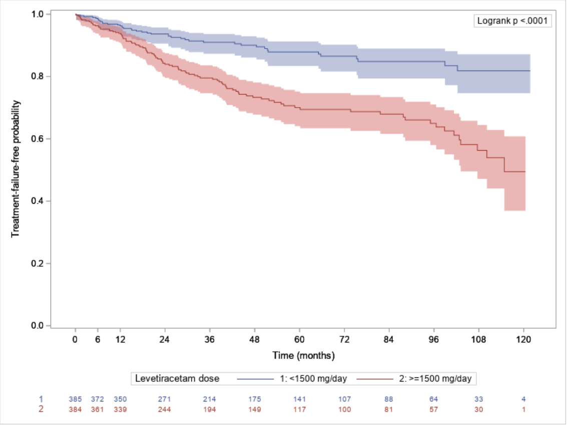

A7

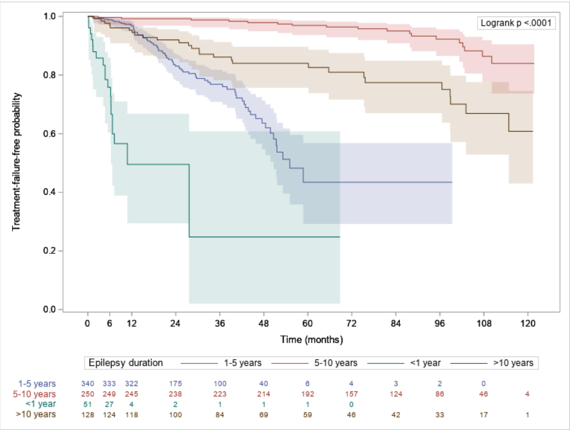
Logrank p <.0001
Treatment-failure-free probability
1.0
0.8
0.6
0.4
0.2
0.0
0 6 12 24 36 48 60 72 84 96 108 120
Time (months)
Epilepsy duration 1-5 years 5-10 years <1 year >10 years
1-5 years 340 333 322 175 100 40 6 4 3 2 0
5-10 years 250 249 245 238 223 214 192 157 124 86 46 4
<1 year 51 27 4 2 1 1 1 0
>10 years 128 124 118 100 84 69 59 46 42 33 17 1

B1

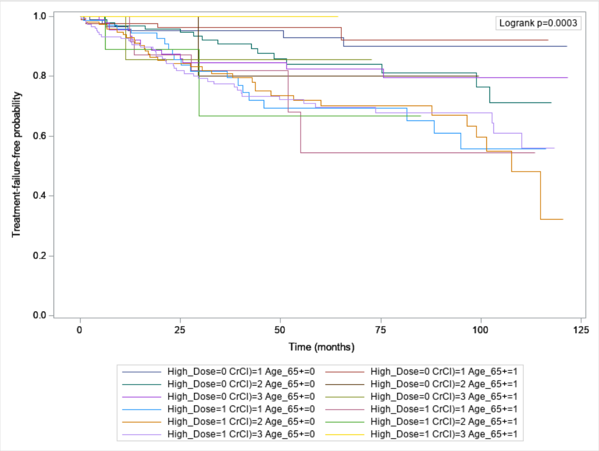
Logrank p=0.0003
Treatment-failure-free probability
1.0
0.8
0.6
0.4
0.2
0.0
0 25 50 75 100 125
Time (months)
High_Dose=0 CrCl)=1 Age_65+=0
High_Dose=0 CrCl)=1 Age_65+=1
High_Dose=0 CrCl)=2 Age_65+=0
High_Dose=0 CrCl)=2 Age_65+=1
High_Dose=0 CrCl)=3 Age_65+=0
High_Dose=0 CrCl)=3 Age_65+=1
High_Dose=1 CrCl)=1 Age_65+=0
High_Dose=1 CrCl)=1 Age_65+=1
High_Dose=1 CrCl)=2 Age_65+=0
High_Dose=1 CrCl)=2 Age_65+=1
High_Dose=1 CrCl)=3 Age_65+=0
High_Dose=1 CrCl)=3 Age_65+=1

B2

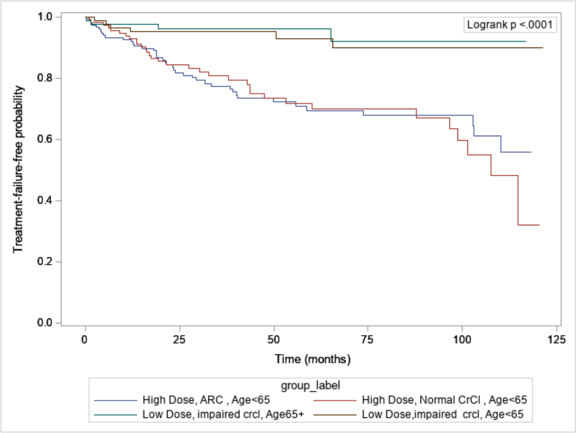
Logrank p <.0001
Treatment-failure-free probability
1.0
0.8
0.6
0.4
0.2
0.0
0 25 50 75 100 125
Time (months)
group_label
High Dose, ARC , Age<65
High Dose, Normal CrCl , Age<65
Low Dose, impaired crcl, Age65+
Low Dose,impaired crcl, Age<65

B1

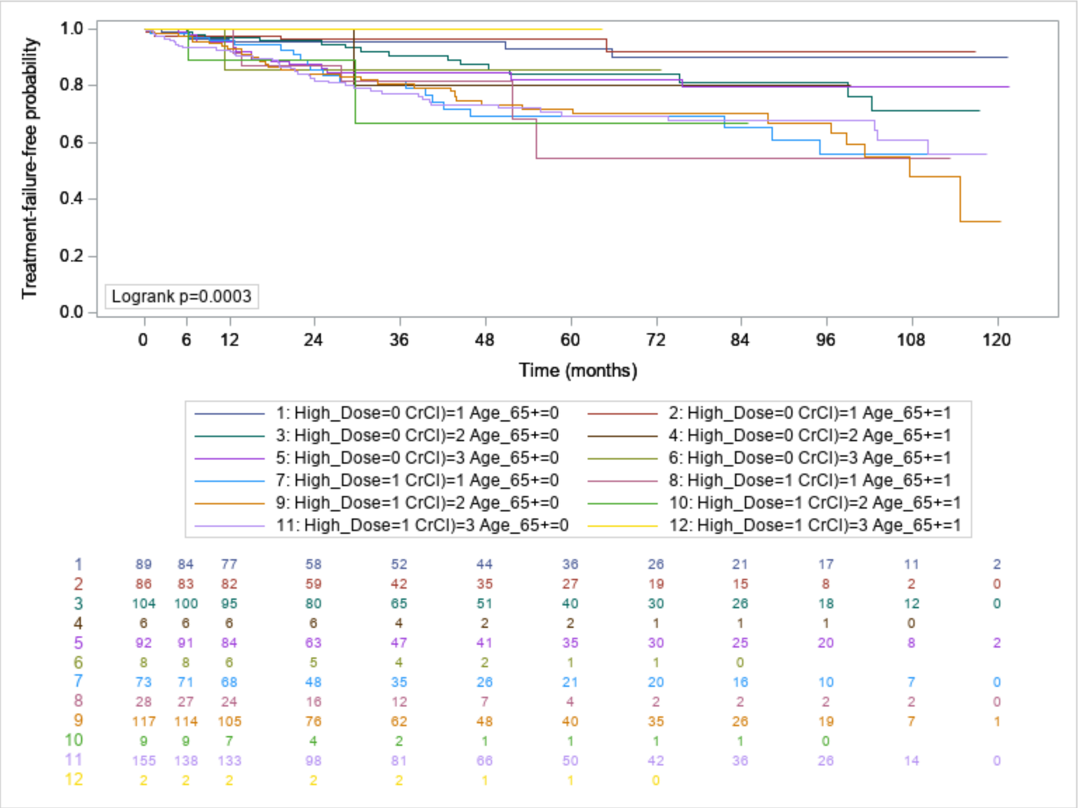

B3

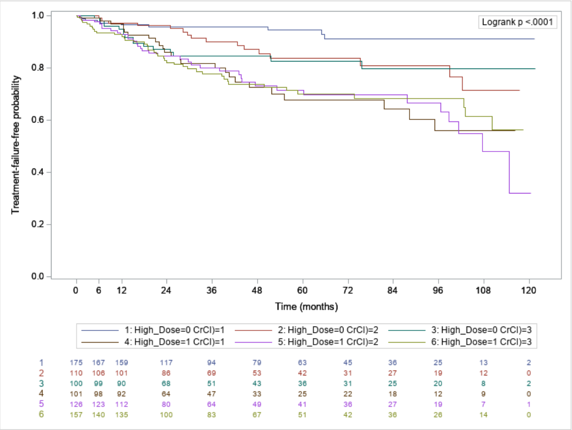

B4

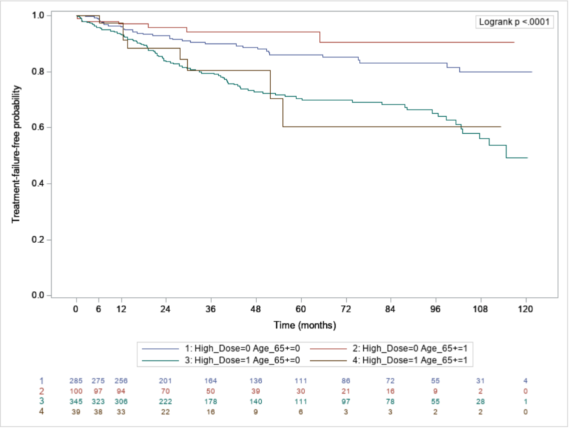

B5

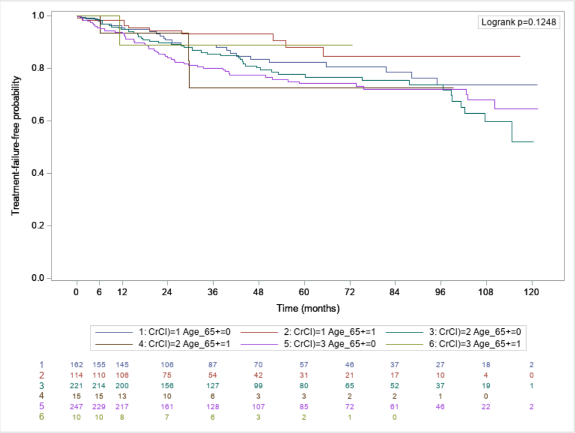

Figure S1. **Kaplan–Meier Subgroup Analyses of Treatment-Failure-Free Probability Across Individual and Combined Strata**. (A) Kaplan–Meier curves stratified by individual patient and clinical characteristics: (A1) age

group, (A2) sex, (A3) seizure onset type, (A4) epilepsy etiology, (A5) creatinine clearance (CrCl) group, (A6) levetiracetam daily dose group, and (A7) epilepsy duration. (B) Kaplan–Meier curves stratified by combined clinical categories: (B1) levetiracetam daily dose, CrCl, and age group; (B2) levetiracetam daily dose and age group; (B3) levetiracetam daily dose and CrCl group; and (B4) age group and CrCl group. Shaded areas represent 95% confidence intervals, and numbers at risk are shown below each plot. Differences between strata were assessed using the log-rank test.
